# Whole-genome sequencing with AVITI and NovaSeq X Plus reveals comparable performance with contextual biases

**DOI:** 10.1093/nargab/lqag053

**Published:** 2026-05-26

**Authors:** Pontus Höjer, Johannes Alneberg, Pär Lundin, Tom Martin, Julia Hauenstein, Helena Fällmar, Magnus Lindell, Christian Natanaelsson, Susana Häggqvist, Adam Ameur, Jessica Nordlund, Robert Månsson Welinder

**Affiliations:** National Genomics Infrastructure, Science for Life Laboratory, KTH - Royal Institute of Technology, Stockholm, 171 65 Solna,Sweden; National Genomics Infrastructure, Science for Life Laboratory, KTH - Royal Institute of Technology, Stockholm, 171 65 Solna,Sweden; National Genomics Infrastructure, Science for Life Laboratory, Stockholm University, Stockholm, 171 65 Solna, Sweden; National Genomics Infrastructure, Science for Life Laboratory, Uppsala University, Uppsala, 752 37 Uppsala,Sweden; Department of Medical Sciences, Uppsala University, Uppsala, 751 85 Uppsala,Sweden; Division of Clinical Immunology, Department of Laboratory Medicine, Karolinska Institute, Stockholm, 141 52 Huddinge,Sweden; National Genomics Infrastructure, Science for Life Laboratory, Uppsala University, Uppsala, 752 37 Uppsala,Sweden; Department of Medical Sciences, Uppsala University, Uppsala, 751 85 Uppsala,Sweden; National Genomics Infrastructure, Science for Life Laboratory, Uppsala University, Uppsala, 752 37 Uppsala,Sweden; Department of Medical Sciences, Uppsala University, Uppsala, 751 85 Uppsala,Sweden; National Genomics Infrastructure, Science for Life Laboratory, KTH - Royal Institute of Technology, Stockholm, 171 65 Solna,Sweden; National Genomics Infrastructure, Science for Life Laboratory, Uppsala University, Uppsala, 752 37 Uppsala,Sweden; Department of Immunology, Genetics and Pathology, Uppsala University, Uppsala, 751 85 Uppsala,Sweden; National Genomics Infrastructure, Science for Life Laboratory, Uppsala University, Uppsala, 752 37 Uppsala,Sweden; Department of Immunology, Genetics and Pathology, Uppsala University, Uppsala, 751 85 Uppsala,Sweden; National Genomics Infrastructure, Science for Life Laboratory, Uppsala University, Uppsala, 752 37 Uppsala,Sweden; Department of Medical Sciences, Uppsala University, Uppsala, 751 85 Uppsala,Sweden; National Genomics Infrastructure, Science for Life Laboratory, KTH - Royal Institute of Technology, Stockholm, 171 65 Solna,Sweden; Division of Clinical Immunology, Department of Laboratory Medicine, Karolinska Institute, Stockholm, 141 52 Huddinge,Sweden; Department of Clinical Immunology and Transfusion Medicine, Karolinska University Hospital, Stockholm, 171 64 Stockholm, Sweden

## Abstract

Element Biosciences’ avidity sequencing has emerged as a competing technology to Illumina’s short-read sequencing platform. Prior benchmarks of avidity sequencing have not included the latest Illumina NovaSeq X/X Plus instruments with XLEAP chemistry. Here, we have run polymerase chain reaction-free whole-genome sequencing on four human tumor cell lines using both Illumina NovaSeq X Plus and Element AVITI instruments. AVITI showed low duplication rates and reported higher base qualities; the latter contributed to improved mapping confidence and fewer spurious variant candidates. Both platforms were found to be highly comparable when benchmarking variant calling, with AVITI only providing a minor improvement on INDELs at lower coverages. Stratifying by genomic context revealed further differences, where AVITI genome coverage and variant calls were superior in high-GC regions while being inferior in GC homopolymers. Error-rate analysis highlighted further differences between the platforms; in particular, AVITI in some instances displayed an increased error rate on read 2 related to short fragments. AVITI error rate was also found to be more stable downstream of repetitive regions, except for GC homopolymers. We further found that AVITI sequencing was sensitive to G-quadruplex motifs. Overall, despite these identified differences, both platforms performed highly comparable for variant analysis.

## Introduction

Massively parallel sequencing has become a true workhorse for genomics research since its introduction in the early 21st century. The ability to read large numbers of DNA sequences has many applications, most prominently in medicine and biology. While initially multiple sequencing technologies competed in this space [1], Illumina’s (Solexa’s) “sequencing by synthesis” [2] rapidly became the dominant technology [3]. Later years have, however, seen increased diversification coupled to the emergence of new competing short-read (<1000 bases) platforms [4], such as Element Biosciences' AVITI system that relies on novel avidity sequencing technology [5].

Avidity sequencing, while similar to Illumina in many regards, comes with several important differences. Both sequencing technologies amplify a single DNA template into ensembles of localized copies on a flow cell surface. These ensembles are then read by incorporating and detecting nucleotide bases one by one along the template, starting from a common primer sequence. Amplification of the single template molecule significantly boosts the signal, facilitating robust detection. For amplification, Illumina relies on polymerase chain reaction (PCR)-like bridge amplification to generate clusters of clonal sequences [2]. Element instead relies on rolling circle amplification (RCA) to generate long strands containing multiple template copies that are collapsed into a structure referred to as a polony [5]. Unlike PCR, RCA avoids propagation of errors since it copies strictly from the original template [6]. For base incorporation and reporting, Illumina uses nucleotides with a fluorescently labeled reversible terminator that are incorporated and imaged before removing the terminator to allow the next base to be incorporated and read [2]. In avidity sequencing, reporting is performed by avidites that are bound, imaged, and subsequently removed to allow incorporation of an unlabeled reversibly terminated nucleotide. Avidites consist of multiple nucleotides of a common type, flexibly chained to a fluorescent core. This setup allows multiple nucleotides from the same avidite to bind complementary bases within the same polony. Binding of the avidites to multiple bases increases the signal retention to improve signal-to-noise ratios [5]. This also limits the impact of phasing, where the reading of an ensemble of template strands lag or advance out of sync to generate a mixed signal [7]. Furthermore, bulky fluorescent labels, as used by Illumina, can exacerbate phasing by impeding base incorporation [8].

Element’s sequencing technology has previously been benchmarked against Illumina for the purpose of whole-genome sequencing (WGS) [5, 9], but to our knowledge, no independent comparison of the technologies has been made. Furthermore, published comparisons were not made to include the latest large-scale sequencing instruments released by Illumina (NovaSeq X/X Plus) using updated X-LEAP chemistry, reported to yield higher base qualities [10]. For this purpose, we performed WGS on four human cell lines, sequencing the same PCR-free libraries on both Illumina NovaSeqX Plus (NovaSeqX+) and Element AVITI instruments. PCR-free libraries were used to limit amplification bias and base errors. Using these datasets, we evaluate multiple characteristics of the technologies for WGS, including variant-calling performance and sequencing error modes in various contexts. Altogether, we find the technologies highly comparable, but with specific differences related to, among other things, genomic context and error modes. Specifically analyzing the rare disparities, we show that Element sequencing is affected by G-quadruplex motifs.

## Methods

### PCR-free library preparation and sequencing

Multiple myeloma (MM) cell lines MM1.S (MM1S), OPM2, and KMS12BM were cultured in RPMI-1640 medium with HEPES and GlutaMAX (Thermo Fisher Scientific, #72400021), supplemented with 10% FCS (Sigma, #F7524) and 100 U/ml Penicillin + 100 μg/ml Streptomycin solution (Cytiva, #SV30010). Genomic DNA (gDNA) was extracted using Monarch® HMW DNA Extraction Kit for Cells & Blood (NEB, #T3050) following the manufacturer’s instructions. The acute lymphoblastic leukemia cell line REH was cultured in RPMI-1640 medium (Sigma, #R0883) supplemented with 2 mM L-glutamine (Sigma, #G7513), 100 U/ml Penicillin and 100 μg/ml Streptomycin (Sigma, #P0781), and 10% heat-inactivated fetal bovine serum (Sigma, #F9665) as previously described [11]. gDNA from REH cells was extracted using the Nanobind CBB Big DNA Kit (Circulomics, NB-900-001-01) following the instructions in “Nanobind UHMW DNA Extraction – Cultured Cells Protocol” (Circulomics, EXT-CLU-001). Library preparation was performed using the TruSeq DNA PCR-Free kit (Illumina, #20015962) according to the manufacturer’s instructions. For each cell line, three replicate libraries were generated, each with unique UDI indexes, resulting in a pool of 12 libraries.

The library pool was sequenced on Illumina NovaSeq X Plus and Element AVITI instruments. Illumina NovaSeq Plus sequencing (performed at the Swedish National Genomics Infrastructure, NGI) was done using two lanes using a NovaSeq X Series 10B 300-cycle Reagent Kit (Illumina, #20085594) with a 2 × 151 base pair (bp) read setup. The lanes were loaded with 140 and 160 pM of denatured library, respectively. The run utilized control software version 1.2.0.28691, and data conversion was done using bcl2fastq version 2.20.0.422. Element AVITI sequencing was initially done using Element Adept Rapid PCR-Free Kit for library conversion, followed by sequencing (performed as a service by Element Biosciences, USA, San Diego) on two Cloudbreak (CB) High Output flow cells (2 × 150 bp, #860-00003). The run was performed using an AVITI instrument, and demultiplexing with bases2fastq v1.6.1.1089765930. Element AVITI sequencing was also done (performed by NGI) using one Cloudbreak Freestyle (CB FS) High Output flow cell (2 × 150 bp, #860-00013) to load the pool without the need for library conversion. Each lane was loaded at 10 and 12 pM, respectively. The run was performed using an AVITI instrument running AVITI OS v2.6.2 and demultiplexing with bases2fastq v1.8.0.1260801529.

### Sequencing data processing

Primary analysis was performed using the nf-core/sarek pipeline (v3.4.2) [12, 13] built on Nextflow (v24.4.2) [14], mapping with BWA MEM (v0.7.17.post1188) [15] to the GRCh38 human reference genome (https://ftp-trace.ncbi.nlm.nih.gov/giab/ftp/release/references/GRCh38/GRCh38_GIABv3_no_alt_analysis_set_maskedGRC_decoys_MAP2K3_KMT2C_KCNJ18.fasta.gz) and marking duplicates with Picard MarkDuplicates (gatk v4.5.0.0, Picard v3.1.1) [16]. As the NovaSeq X Plus reads were one base longer than the AVITI, the pipeline was configured to cap reads to the same 150 bp using fastp (v0.23.4) [17] while skipping the default adaptor, quality, and polyG trimming. Besides capping reads to 150 bp, no modification or filtering of the reads were performed. Base recalibration was further disabled (option “--skip-tool baserecalibrator”) to preserve the original base qualities. All steps described earlier were applied identically to all AVITI and NovaSeq X Plus datasets. Any subsequent data reduction steps (e.g. downsampling or restriction to specific genomic regions) were performed only for specific downstream analyses and are described in the corresponding sections.

Information about insert size, base qualities, and mapping quality (MAPQ) was gathered from samtools stats (v1.19.2) [18], which is part of the nf-core/sarek pipeline.

### Duplicate reads investigation

Reads marked as duplicates by GATK/Picard MarkDuplicates were investigated to see if the origin could be optical or from complementary strands. Optical duplicates are physically proximal on the flowcell and originate from either (A) one template cluster/polony falsely detected as separate clusters/polonies or (B) one template (or sequencing-derived copies thereof) seeding multiple clusters/polonies. Complementary duplicates originating from opposing strands of the same double-stranded DNA fragment can seed independently on the same flowcell and falsely be marked as duplicates; this is also called “sister duplicates.” Analysis of duplicates was limited to reads mapped to chr20 for computational efficiency. For this, reads were remarked with MarkDuplicates but with the option “--TAG_DUPLICATE_SET_MEMBERS true” to label reads involved in a duplicate set. These labeled reads were then extracted using samtools view with options “-d DI --keep-tag DI,DS,RG” and investigated using a custom script (duplicates.py), running Python (v3.12.7) with the pysam (v0.22.1) package. For each combination of read pairs in a duplicate set, the distance between the pairs and the pair’s relative read1/2 order was computed. The Euclidean distance between duplicate read pairs were calculated using the reads cluster/polony coordinates (tile, x-position, y-position) encoded in the sequence identifier. Note that the distance units are dimensionless, as they refer to flowcell pixel locations that are instrument dependent. Pairs within 100 distance units on the sample flowcell tile were classified as “proximal.” Pairs where the reads map in opposite directions were classified as “complementary.” Each combination of classifications were tallied.

### Differential coverage

GRCh38-mapped reads from all datasets were first randomly downsampled to 10× coverage to ensure comparable effective coverage. Downsampling was performed using samtools view (v1.19.2) with option “-s {fraction}.” The fractions were based on the mean coverage acquired using mosdepth (v0.3.8) [19]. Files in d4 format [20] were generated using mosdepth (v0.3.10) to look up the coverage per base across multiple genomic regions. To avoid mismapped reads, we only included reads with a MAPQ of at least 60. The d4 file format allows for fast coverage queries across multiple intervals. To assay coverage across different genomic contexts, we used the Genome in a Bottle (GIAB) GRCh38 genome stratifications (v3.5, https://ftp-trace.ncbi.nlm.nih.gov/giab/ftp/release/genome-stratifications/v3.5/) [21] with d4tools (v0.3.10 with d4 library v0.3.9) to get coverage distributions for each stratification. The mean coverage for each stratification and sample was calculated from the distribution. Stratifications covering <10 000 bases across the genome were excluded from further analysis to avoid unstable measurements. We further excluded stratifications in the subcategories “Ancestry,” “FunctionalTechnicallyDifficult,” and “GenomeSpecific” as these were not considered as informative for this analysis. For each stratification, the relative coverage difference to the overall autosomal coverage (stratification “GRCh38_AllAutosomes.bed.gz”) was calculated. To focus on differences between the sequencing technologies, we selected the 15 most variable stratifications based on variance in mean coverage across the AVITI and NovaSeqX+ runs for each cell line.

### PacBio HiFi sequencing for variant benchmarking

PacBio HiFi sequencing libraries for MM1S, OPM2, and KMS12BM were prepared using the same high-molecular-weight gDNA source as the short read libraries and the SMRTbell prep kit 3.0 (Pacific Biosciences, #102-141-700). Sequencing was done on the PacBio Revio system. The PacBio reads were mapped to GRCh38 using pbmm2 (v1.10.0, https://github.com/PacificBiosciences/pbmm2). Sequence length and base quality was assessed using sequali (v1.0.2) [22]. Genome coverage was computed using mosdepth (v0.3.8). Small variants were called using DeepVariant (v1.5) using the “PACBIO” model. For generation of high-confidence regions, structural variants were called using PBSV (v2.9.0, https://github.com/PacificBiosciences/pbsv) and copy number variations (CNVs) using HiFiCNV (v0.1.7-70e9988, https://github.com/PacificBiosciences/HiFiCNV) with option “--exclude cnv.excluded_regions.common_50.hg38.bed.”

### Variant calling and benchmarking

Element and Illumina small variants were called with DeepVariant (v1.5.0) [23] using the model “WGS.” The analysis was limited to autosomal chromosomes. Read were randomly downsampled to 10×, 15×, 20×, 25×, 30×, 40×, and 50× using DeepVariant with the option “–make_examples_extra_args downsample_fraction={fraction}” using fractions calculated from the mean coverage acquired from mosdepth (v0.3.8).

Benchmarking was performed using hap.py (v0.3.15) [24] with the RTG-tools vcfeval (v3.12.1) engine with option “--engine = vcfeval.” PacBio DeepVariant PASS calls were used as truth set, and with option “--preprocess-truth” enabled. Benchmarking was limited to high-confidence regions (see section below for how these were generated). To query performance in different genomic contexts, GIAB stratifications (v3.5) were used. Stratifications covering <500 PacBio variants were excluded to avoid unstable metrics. Similar to the differential coverage analysis, we excluded some stratifications not deemed relevant, such as the subcategories “Ancestry” and “GenomeSpecific.” Variant-calling performance was assessed using the F1-score metric calculated as below.


\begin{eqnarray*}
F1\ \mathrm{score}\ = \ \frac{{T{{P}_{\mathrm{Truth}}} + T{{P}_{\mathrm{Query}}}}}{{T{{P}_{\mathrm{Truth}}} + T{{P}_{\mathrm{Query}}} + FP + FN}}.
\end{eqnarray*}

Here, true positives ($TP$) are disambiguated into $T{{P}_{\mathrm{Truth}}}$, the number of variants in the truth-set that match the query, and $T{{P}_{\textit{Query}}},$ the number of variants in the query that match the truth-set. False positives ($FP$) are query variants not matched in the truth-set. False negatives ($FN$) are truth-set variants that are missing from the query.

### Generation of high-confidence benchmarking regions

We generated high-confidence benchmarking regions for each MM cell line, focusing analysis on autosomal chromosomes. First, across all datasets, we excluded regions with excessively low or high coverage from the benchmarking regions. For this purpose, mosdepth (v0.3.8) was used to query the coverage in 100 bp windows, limiting to reads with at least MAPQ 60 for Illumina/Element and MAPQ 20 for PacBio. Any windows with <10× coverage or more than two times the median dataset coverage were excluded. The remaining Illumina/Element regions were merged using bedtools (v2.30.0) [25] for each cell line. These regions were then intersected with the corresponding PacBio regions.

Regions containing clustered variants were excluded from the benchmarking regions to avoid ambiguity in small-variant evaluation. Complex small-variant regions were excluded by finding regions with two or more small variants within 10 bp in the filtered PacBio DeepVariant calls and extending these by 50 bp on each side. We also excluded regions within 50 bp of structural variants >50 bp called with PBSV using the PacBio data.

DeepVariant is designed for diploid genomes. We therefore excluded regions containing CNVs that may confound germline variant calling. For this, PacBio HiFiCNV calls were expanded by 1000 bases and excluded for the high-confidence regions.

Following benchmarking practices from the GIAB consortium [26, 27], we further excluded contigs <500 kb (GRCh38_contigs_lt500kb) and regions close to reference gaps (GRCh38_gaps_slop15kb), relying on GIAB stratifications v3.5. We further excluded regions with long homopolymers (GRCh38_SimpleRepeat_imperfecthomopolge11_slop5 and GRCh38_SimpleRepeat_homopolymer_ge12_slop5) that are problematic for PacBio HiFi reads [28, 29]. Finally, partially covered tandem repeats (TRs) or homopolymers (defined by GRCh38_AllTandemRepeatsandHomopolymers_slop5) were excluded, similar to Wagner *et al*. [27]. Bedtools (v2.30.0) [25] and bcftools (v1.18) [18] were used extensively for generating the high-confidence regions.

### Publicly available avidity sequencing datasets

For the error rate evaluation, we, in addition to our own data, utilized publicly available Element avidity sequencing data, generated using different PCR-free library preparation kits and AVITI flowcells (Supplementary Table S1). For five datasets, we downloaded BAM files with GRCh38-mapped reads. The HG002 Cloudbreak Freestyle dataset, which was only available as FASTQ files, was downloaded and mapped to GRCh38 using the nf-core/sarek pipeline (v3.4.2) and the same configurations as described earlier.

### Error rate investigation

Except for error rate investigations in different genomic contexts, we limited analysis to chr20 for computational efficiency, and is largely representative of the human genome. Furthermore, only reads with a mapping quality of at least 60 were included to minimize the inclusion of mismapped reads.

Reference-based error rate per cycle was evaluated using samtools (v1.19.2) for read 1 and read 2 separately. Reads were selected using samtools view, and statistics were calculated using samtools stats. Bases and substitutions versus the reference were aggregated for each cycle to calculate the error rate. The error rates were smoothed using a 5 bp rolling average across cycles.

We evaluated the overlap-based error rate per cycle using fraguracy (v0.2.4, https://github.com/brentp/fraguracy) with the option “--bin-size .1”. The error rates were smoothed using a 5 bp rolling average across cycles. To calculate the median error rate per read, only bases at positions 50%–60% into the read were considered, similar to Stoler & Nekrutenko [30].

For the insert-size based error evaluation, reads were selected by their template length in bins of 50 bp using samtools view for read 1 and 2 separately. Bases and errors versus the reference were calculated using samtools stats as earlier.

To evaluate error rate by genomic context, we relied on the “stack_reads_by_interval.py” script from Arslan *et al*. (commit 701be39, https://github.com/Elembio/AvidityManuscript2023) [5]. Repetitive regions in GRCh38 we acquired from GIAB (v3.5). A control set of 30 bp random regions was generated using bedtools random with options “-l 30.” A maximum of 10 000 regions across all chromosomes was randomly picked for each stratification to limit the computational load but still cover a large number of regions. Briefly, reads with MAPQ ≥ 60 overlapping the regions were extracted using samtools. These reads were then piled up across each region by strand to identify substitutions versus the reference using the “stack_reads_by_interval.py” script. The script computes the error rate for the bases upstream, inside and downstream of the regions. For the upstream versus downstream error rate change plots, only the 50 bases closest to the region were used.

### G-quadruplex investigation

The G-quadruplex (G4) investigation relied on predicted G4 (pG4) motifs generated using pqsfinder (v2.0.1) [31] with default options on GRCh38 downloaded from https://pqsfinder.fi.muni.cz/genomes.

To study the overlap between extensively soft-clipped alignments and pG4 motifs, we extracted reads with at least 10 soft-clipped bases using samtools (v1.19.2). Only autosomal reads with a MAPQ greater than or equal to 60 and a template length (insert size) >150 bp were included. These reads were converted to BED format using bedtools (v2.30.0) bamtobed and split by strand information (forward or reverse). For each strand and sample, the read spans were extended by 10 bp on the ends using bedtools slop and then merged using bedtools merge, counting the number of overlapping spans. Only spans with at least two soft-clipped reads were kept. For each strand and cell line, only spans shared across the AVITI CB and AVITI CB FS runs were kept. These spans were further filtered to only keep those that were shared across at least two cell lines. This yielded locations of prominent soft-clipping across the AVITI datasets on each strand. Soft-clipping is common for reads that overlap structural variations. To exclude such sites, locations with overlapping soft-clipped reads on both strands were removed. As an additional layer of filtration, common soft-clipped sites in the NovaSeq X Plus datasets were subtracted from the AVITI sites to get sites specific to the AVITI data. Overlap between the AVITI soft-clipped sites and pG4 sites by strand was queried using bedtools intersect.

We further looked at base quality scores and mismatched bases for reads overlapping pG4 sites. G4 sites might be proximally spaced, leading to a read spanning multiple sites. Therefore, we focused on isolated pG4 sites, removing any sites within 300 bp of each other using bedtools (v2.30.0). Any pG4 sites >50 bp were also excluded to only include sites that can be covered by a 150 bp read. Analysis was limited to pG4 sites on chr20 for computational efficiency. Reads overlapping the isolated pG4 sites were extracted using samtools (v1.19.2). Only reads that covered the pG4 sites by at least 5 bp on each side were included. Both the isolated pG4 sites and the overlapping reads were split by strand. Reads were piled up for each site expanded by 150 bp using samtools mpileup with options “--no-BAQ --min-BQ 0 -a -x.” From the pileups, base qualities and mismatched bases were collected for each position relative to the pG4 site on both strands using a custom Python script.

The error rate following G4 motifs was assayed using the stack_reads_by_interval.py script from Arslan *et al*. (commit 701be39, https://github.com/Elembio/AvidityManuscript2023) [5], similar to the error rate investigation detailed earlier.

### Data visualizations

Data visualizations were generated using Python (v3.12.7) with extensive use of packages Jupyter (v5.7.2) [32], seaborn (v0.13.2) [33], pandas (v2.2.3) [34, 35], matplotlib (v3.9.2) [36], ​NumPy (v2.1.3​) [37], and SciPy (v1.14.1) [38]. Figures and illustrations were composed and visually adjusted for clarity using Affinity Designer (v2.6.0).

## Results

**Figure 1.**
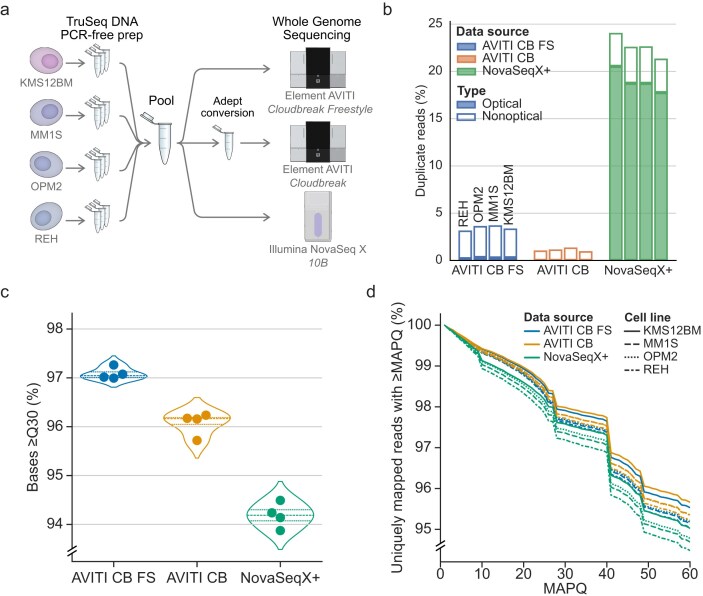
Comparative performance of Element and Illumina NovaSeq X Plus for WGS. (**a**) Study design. (**b**) Duplication rates by category. For the Illumina NovaSeq X Plus results, it should be noted that one lane had an overall inflated duplication rate due to suboptimal loading, see Supplementary Table S2. (**c**) Reported base quality greater than or equal to Q30. (**d**) Mapping confidence for BWA-MEM on GRCh38 as measured by the percentage of all uniquely mapped reads with a particular mapping quality (MAPQ) or greater.

To directly compare Element to Illumina sequencing, the same pool of PCR-free gDNA libraries was sequenced on both the Element AVITI and the Illumina NovaSeq X Plus sequencers (Fig. 1a). Illumina-style PCR-free WGS libraries were prepared using DNA from four cell lines, three of MM origin (MM1S, OPM2, KMS12BM) and one with acute lymphocytic leukemia origin (REH). For Illumina sequencing, the pool was loaded on two 10B flowcell lanes on a NovaSeq X Plus instrument. Two rounds of Element AVITI sequencing were performed. In the first round (referred to as AVITI CB), libraries were converted to circular Element-style libraries and loaded onto two CB flow-cells for sequencing. In the second round (referred to as AVITI CB FS), libraries were directly loaded on a single Element CB FS flowcell for on-board circularization and sequencing. A schematic overview of the different workflows can be seen in Supplementary Fig. S1.

Initial analysis and quality control of all demultiplexed reads from the instruments were performed using the nf-core/sarek pipeline (v3.4.2). As the NovaSeq X Plus reads were one base longer than the AVITI, all reads were trimmed to the same 150-base length within the pipeline. All reads were then mapped to GRCh38 using BWA-MEM, and duplicates marked with GATK/Picard MarkDuplicates. No read filtering, additional trimming, or manipulation was performed. Unless explicitly stated otherwise, all reported metrics were computed using the full mapped datasets. Mapping rates were high (>99%) across all samples (Table 1). Insert sizes were mostly similar across all datasets (∼400 bp), apart from AVITI CB FS inserts being on average ten bases shorter (Supplementary Fig. S2). The cause for this fragment selection is currently unknown but may relate to the on-board circularization. Mean coverage was expectedly lower for the AVITI CB FS datasets (15.2 to 20.0×) since only a single flowcell was run, while the other datasets had a mean coverage above 40× (Table 1).

**Table 1.** AVITI and NovaSeq X Plus statistics for cell line libraries and sequencing setups. CB = Cloudbreak, FS = Freestyle

Data source	Cell line	Read pairs (M)	Mean insert size (bp)	Mapped (%)	MAPQ = 0 (%)	Mean coverage (×)
AVITI CB FS	KMS12BM	190	392	99.9	4.0	17.7
AVITI CB FS	MM1S	191	397	99.9	4.2	17.7
AVITI CB FS	OPM2	216	387	99.9	4.1	20.0
AVITI CB FS	REH	163	410	99.8	4.1	15.2
AVITI CB	KMS12BM	539	406	99.9	3.7	51.4
AVITI CB	MM1S	525	411	99.9	3.9	49.9
AVITI CB	OPM2	526	400	99.9	3.9	50.1
AVITI CB	REH	475	425	99.9	3.9	45.3
NovaSeqX+	KMS12BM	694	406	99.7	4.6	52.4
NovaSeqX+	MM1S	687	412	99.7	4.9	51.0
NovaSeqX+	OPM2	787	400	99.7	4.8	58.4
NovaSeqX+	REH	641	425	99.7	4.9	46.7

High duplication rates reduces effective coverage and increases sequencing cost. Increased duplication rates have been reported for other Illumina instruments compared to Element avidity sequencing [5, 39]. Unlike Element, Illumina clustering generates multiple copies of the original fragment that can seed adjacent wells in patterned flow cells to generate optical duplicates. Indeed, NovaSeq X Plus duplication rates were found to be higher compared to the AVITI runs and primarily driven by optical duplicates (Fig. 1b). In our results, the NovaSeq X Plus duplicate rates were partially inflated by nonoptimal loading in the lane with the lower loading concentration (140 pM) (Supplementary Table S2). However, even in the best NovaSeq X Plus lane, duplicates contribute to a substantial portion of reads (12%–13%) (Supplementary Table S2). Element AVITI duplicates were low (<5% in all lanes) and primarily non-optical. Further inspection revealed AVITI duplicates were misclassified, with ∼90% of duplicate-marked AVITI read pairs originating from sequencing of the complementary strands of the original gDNA fragment (Supplementary Fig. S3). These reads marked as non-optical duplicates were also slightly less frequent in AVITI CB than AVITI CB FS, possibly due to the CB library conversion that denatures and dilutes complementary strands before loading. Hence, the actual duplicate rate in AVITI was very low (<0.5%) compared to NovaSeq X Plus. Based on this observation, duplicate marking may be inadvisable for AVITI sequencing of PCR-free libraries.

Sequencing instruments generally report a Phred quality score (Q) for each called base that estimates the probability of the base being called correctly. Avidity sequencing has been reported to give highly accurate base calls, with 85.4% of bases being > Q40 (less than one error per 10 000 bases) [5]. Similar values were found in the AVITI CB datasets reported here, with 84.0%–85.4% of base qualities > Q40. The AVITI CB FS base qualities were even higher, with 87.7%–88.5% of bases > Q40. Direct comparison to Illumina NovaSeq X Plus data is made complicated by the instrument reporting Q scores in four coarse bins (bin 2, Q0-2; bin 12, Q3-17, bin 24, Q18-29; bin 40, Q30+; with Control Software v1.2.0), where base qualities ≥ Q30 are reported in bin 40 (Supplementary Fig. S4). We therefore relied on the fraction of bases ≥ Q30 for comparisons against the AVITI datasets. The AVITI datasets had 95.7%–97.3% of bases ≥ Q30, compared to ∼94% for NovaSeq X Plus data (Fig. 1c). In the AVITI data, the Q-scores were also slightly higher in the AVITI CB FS datasets, which may be due to the lower polony density (∼800 million per lane) compared to the AVITI CB runs (∼1000 million per lane).

### Comparison of mapping confidence, coverage biases, and variant calling

To evaluate the effects of the higher-accuracy base calls in the AVITI data, we investigated alignment to the GRCh38 human reference genome. Sequencing errors complicate genome mapping, as they increase the number of plausible locations within the reference. Most mappers, including BWA-MEM, report a phred-scaled mapping quality (MAPQ) that measures the certainty of the read placement. A MAPQ ≥ 60 corresponds to an estimated mismapping probability below one in a million. Reads that are equally likely to map to multiple locations are given a MAPQ of zero. This was more commonly observed for NovaSeq X Plus reads (4.6%–4.9% of mapped reads) compared to AVITI (3.7%–4.2% of mapped reads). For uniquely mapped reads, the AVITI datasets generated higher MAPQ scores with BWA-MEM relative to NovaSeq X Plus (Fig. 1d). These findings indicate that the increased base-call accuracy of AVITI data contributes to improved read mapping confidence.

**Figure 2.**
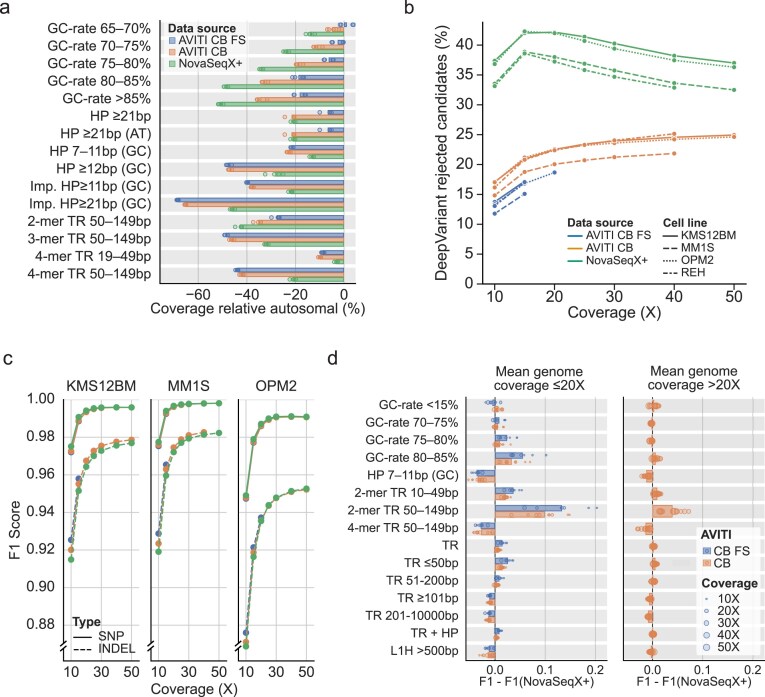
Comparisons of genome coverage and variant calling. (**a**) Differential coverage across the top 15 most variable genome stratifications. Difference measure relative to mean autosomal coverage for the dataset. (**b**) Candidate small variant calls filtered out by DeepVariant across coverage from 10× to 50×. (**c**) F1-score for variant calling for SNPs and INDELs at different levels of genome coverage, as reported by hap.py. PacBio HiFi DeepVariant calls were used as the truth set. Cell line REH was omitted as HiFi coverage was insufficient. (**d**) Relative F1-score for SNPs and INDELs combined comparing AVITI to NovaSeq X Plus across GIAB stratifications (v3.5). The plot shows the top 15 most variable genome stratifications, split by mean overall coverage range. Bars show the mean difference across the subsampled datasets. TR = tandem repeat, HP = homopolymer, Imp. HP = imperfect HP, N-mer = TR composed of N bp repeated elements.

Looking further into the alignments, we investigated coverage biases by genomic stratifications. Genomic stratifications that partition the genome into regions with shared properties, such as high-GC content or repeat structures, were acquired from GIAB [21]. To measure the coverage bias, all datasets were downsampled to 10× coverage, and the difference in mean coverage per stratification to the autosomal coverage was calculated for each sample. Looking at the top variable stratifications, most coverage variation was observed in repetitive and high-GC regions (Fig. 2a). Compared to NovaSeq X Plus, the AVITI samples had higher coverage in high GC regions and 2-mer TRs between 50 and 149 bp, while coverage was lower in 3- and 4-mer TRs as well as GC homopolymers (HPs) and GC imperfect HPs. AVITI CB and CB FS displayed mostly similar coverage profiles, but AVITI CB FS had higher coverage in long (≥21 bp) non-GC homopolymers (HPs) and in high-GC regions.

Besides mapping, base quality also influences variant calling. To investigate this, we used the germline variant caller DeepVariant [23] v1.5, which includes a model trained on both Illumina and Element sequencing data. DeepVariant generates candidate variant calls, which the model then evaluates to either reject or keep the call. Candidate generation is highly permissible to increase sensitivity, requiring support of two reads as well as allele frequencies of at least 6% (SNPs) or 12% (INDELs). Carrol *et al*. found substantially more rejected candidates in Illumina NovaSeq 6000 data compared to Element AVITI [9]. We similarly found that the proportion of rejected candidates was higher in Illumina NovaSeq X Plus across a wide range of coverages (Fig. 2b). This implies that there is a significant portion of non-random sequence errors that appear as putative variants in the NovaSeq X Plus data. While these were rejected by DeepVariant as germline variants, they could possibly impede somatic variant calling.

We further evaluated the accuracy for the DeepVariant calls that passed the model evaluation. The Element AVITI variant calls were previously found to be more accurate compared to Illumina NovaSeq 6000, especially at lower genome coverage [9]. To benchmark the variant calls, we compared them against an orthogonal sequencing technology. For this, we used high-accuracy PacBio HiFi long-read data generated on the Revio platform from the same gDNA source for the MM1S, OPM2, and KMS12BM cell lines (Supplementary Table S3). REH was not used in this analysis, as existing PacBio data were low coverage and generated using the Sequel II instrument [11]. Using the MM1S, OPM2, and KMS12BM PacBio data, DeepVariant-called variants were used as a reference set for benchmarking the Element and Illumina variant calls. While this reference is not an absolute truth set, it should nevertheless enable us to identify major platform differences. Variant-call benchmarking was limited to a set of high-confidence regions to assess false-positive calls (see Methods for details) [24]. Briefly, these regions remove extreme low/high coverage regions that could influence comparisons. Following previous benchmarking efforts by the GIAB consortium [26, 27], these regions also exclude short reference contigs as well as regions close to genome gaps, complex variants, and variants overlapping structural variants or CNVs. Long homopolymers were excluded as they are problematic for PacBio sequencing [28, 29]. Variant calling performance was measured using the F1-score, which is the harmonic mean of recall and precision. Overall, our analysis showed that the variant-calling performance between AVITI and NovaSeq X Plus was highly comparable (Fig. 2c) with AVITI only providing slightly higher F1-scores for INDELs at low coverage, particularly in the AVITI CB FS data.

Investigating these variant calls further, we looked at the performance by genomic context for SNPs and INDELs combined. For annotations on genomic context, we again relied on GIAB stratifications [21]. Similar to the coverage analysis, most variation relative to NovaSeq X Plus in variant-calling performance was seen in shorter repetitive regions and regions with extreme GC rates (Fig. 2d). Most noticeably, the AVITI runs had increased F1-score for short (<50 bp) TRs, high (75%–80%) GC regions, and specifically for longer (50–149 bp) 2-mer repeats. The AVITI F1-score was lower in 7–11 bp GC homopolymers and longer repeats, in particular for longer (50–149 bp) 4-mer TRs. This pattern largely follows the coverage differences between AVITI and NovaSeq X Plus identified earlier. Furthermore, at higher (>20×) coverage, the F1 difference is somewhat muted, reinforcing the influence of coverage.

### Exploring sequencing error modes

**Figure 3.**
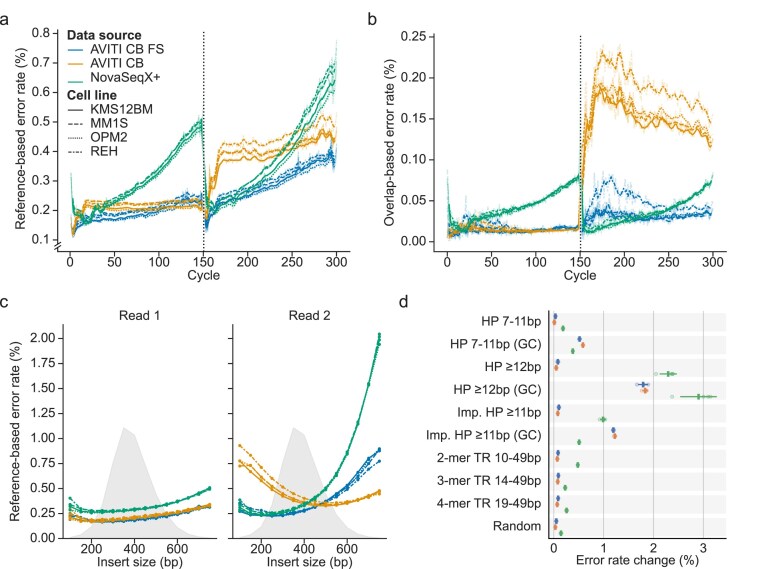
Variations in error rate in the form of substitutions across datasets. Error rate across cycles based on (**a**) difference to reference and (**b**) read overlap disagreement. The dotted line shows the boundary between reads 1 and 2. (**c**) Reference-based error rate across insert sizes. Insert sizes calculated in 50-bp bins. The gray area shows the overall read density for each bin. (**d**) Change in error rate following small repeat regions for overlapping reads. Repeats regions acquired from GIAB stratifications (v3.5). A set of random 30-bp regions (Random) was also included for reference. Reference-based error rate computed for reads overlapping small repeats. Error rate change was calculated for the 50 bp downstream of the region relative to the 50 bp upstream. Repeats composed solely of G or C bases are labeled GC. HP = homopolymer, Imp. = Imperfect, TR = tandem repeat.

We further investigated the sequencing error rate to evaluate the accuracy of base calls in the different samples. First, we looked at differences in the individual read compared to the reference genome. This error measure is not absolute, as it also includes true variants reported as errors, but it can still highlight differences between the runs. The majority of mismatches were substitutions, making these the focus for the continued investigation (Supplementary Fig. S5). Looking across cycles and reads, the NovaSeq X Plus displays a significant increase in reference-based error rate toward the end of each read (Fig. 3a). A similar pattern has been observed for other Illumina instruments [40]. In the AVITI run, the error rate is mostly stable across cycles, but considerably higher in read 2 for AVITI CB compared to AVITI CB FS. A similar pattern of increased error rate in read 2 could also be observed in publicly available AVITI datasets (Supplementary Fig. S6a and b).

Instead of relying on the reference, an absolute measure of the sequencing error rate can be computed by investigating read pair overlaps [30]. Read pairs with short inserts have overlapping sequences that can be compared to identify errors introduced in sequencing. Importantly, these errors can be identified independently of true variants or pre-sequencing artifacts, e.g. caused by DNA damage. Stoler & Nekrutenko estimated the overlap-based error rate in the middle of the read for Illumina NovaSeq 6000 to a median value of 0.109% [30]. For NovaSeq X Plus, this rate was estimated to be between 0.036% (OPM2) and 0.040% (REH), which is considerably lower as compared to NovaSeq 6000 rates. Using this error-rate measure, the pattern of increased error toward the later cycles remains for NovaSeq X Plus, although with a lower rate overall (Fig. 3b). Notably, we observe a jump in error rate for read 2 in the AVITI CB data (Fig. 3b). A similar jump was also observed in one of the public AVITI CB datasets (Supplementary Fig. S6C). Apart from this, we see the same trends as in the reference-based analysis, with a lower read1 error rate for AVITI.

Overlap-based error profiling is limited to pairs with inserts shorter than the combined pair read length (300 bp in this study). To investigate whether insert size could bias the observed jump in error rate for read2 in the AVITI CB, we profiled the reference-based error rate across insert sizes (Fig. 3c). The AVITI CB sample had a clear pattern of increased error rate for short inserts in read2, which would explain the sudden increase in the overlap-based error rate (Fig. 3b). A similar trend was also visible in the public AVITI CB dataset that displayed a similar jump (Supplementary Fig. S6d). Investigating the AVITI CB error-rate increase for short inserts in read 2, we looked at the bases involved in the errors and found that the read 2 errors were primarily C > T/G > A transitions followed by C > A/G > T (Supplementary Fig. S7). The same pattern could not be observed for the NovaSeqX+ and AVITI CB FS runs, where instead the error rate increased with larger insert sizes (Fig. 3c). In particular, the NovaSeq X Plus samples had substantially increased error rate for ≥500 bp inserts in read 2, similar to what has been observed with other Illumina instruments [41]. Overall, this result highlights the benefits of a narrow fragment size distribution when preparing libraries for NovaSeq X or AVITI sequencing.

### Influence of repetitive regions on read error rate

Sequence context, and in particular homopolymers, is known to influence error rates in Illumina sequencing [30, 42]. To investigate this further, we evaluated error rates in reads spanning repetitive regions as defined by the GIAB [21]. Specifically, we compared changes in error rates for bases downstream of these regions. Previous studies have shown that AVITI sequencing exhibits lower error rates downstream of long homopolymers than Illumina NextSeq 2000 and NovaSeq 6000 [5, 43]. Our data show that Illumina NovaSeq X Plus has a substantially increased error rate downstream of homopolymers and tandem repeats, while AVITI maintains a relatively stable error rate in those contexts (Fig. 3d). However, across GC homopolymers, AVITI shows a notable error-rate increase, often exceeding that of the NovaSeq X Plus data. These elevated error rates may lead to false-positive variant calls, potentially contributing to reduced F1-scores observed in GC homopolymers of 7–11 bp in length (Fig. 2d). While this highlights a potential bias in the avidity sequencing, the impact is likely limited, as such GC homopolymers comprise a small fraction of the human genome (Supplementary Table S4).

### G-quadruplex motifs influence AVITI sequencing

**Figure 4.**
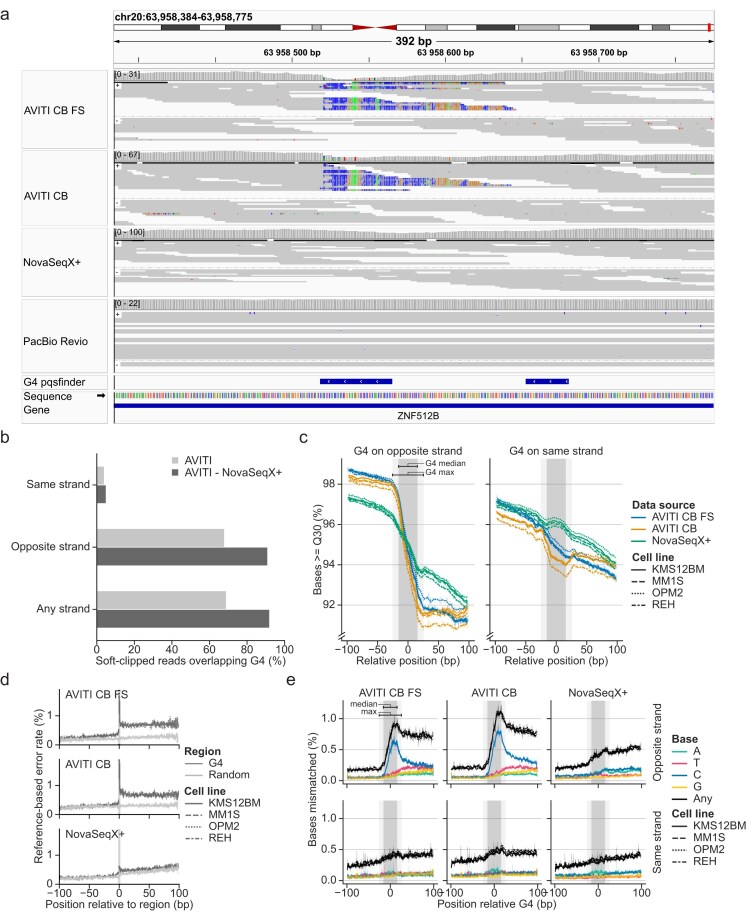
G-quadruplex (G4) related errors in Avidity sequencing. (**a**) IGV (v2.16.2) [49] example from MM1S cell line. (**b**) AVITI soft-clipped reads overlap predicted G4 regions on the opposing strand. (**c**) G4 regions cause a drop in reported base quality that persists to downstream cycles. The gray region shows the median (inner) and maximum (outer) G4 span. (**d**) G4 regions cause an increase in error rate. (**e**) Erroneous base calling around G4 regions causes an abundance of cytosine calls.

Inspection of the datasets revealed frequent and strand-specific soft-clipping at shared locations among AVITI datasets not found in data from the other instruments (Fig. 4a). Soft-clipping is a common alignment strategy used to improve alignment accuracy by omitting bases from the ends of reads that significantly diverge from the reference sequence. Notably, many of these soft-clipped loci appeared to colocalize with G-quadruplex (G4) motifs. G4 motifs can form noncanonical DNA structures composed of four guanine repeats interspersed with short loop sequences of other bases that can fold into a stable secondary structure [44, 45]. These motifs are common in the human genome, with one recent study finding ∼700 000 G4 motifs [46]. Because G4 structures form on one strand, their impact is inherently strand dependent.

To explore the relationship between soft-clipping in AVITI samples and G4 motifs, we used predicted G4 regions for GRCh38 from pqsfinder [31]. Reads with ≥10 soft-clipped bases were intersected by strand across AVITI datasets to identify common clipping sites. Sites overlapping across strands were excluded, and further filtering removed any commonly soft-clipped loci present in the NovaSeq X Plus data to remove shared structural variants or other artifacts across cell lines. This analysis revealed that the majority of recurrent AVITI soft-clipped sites overlapped predicted G4 motifs, and these overlaps were almost exclusively strand-specific, occurring on opposite strands (Fig. 4b). Possibly, this can partially explain the biases observed for AVITI in and around GC homopolymers (Figs 2a, d and 3d), as these often colocalize with G4 motifs (Supplementary Table S5).

The recurrent soft-clipping of AVITI reads overlapping G4 motifs on opposite strands suggests that avidity sequencing accuracy may be reduced in regions surrounding these noncanonical DNA structures. G4 motifs have been associated with increased error rates across multiple sequencing platforms [47, 48]. To investigate this further, we analyzed base quality scores for reads overlapping predicted G4 motifs, stratified by strand. In AVITI samples, we observed a pronounced decrease in base quality following G4 motifs on the opposite strand (Fig. 4c), whereas NovaSeq X Plus showed only a modest decline. Interestingly, base quality before G4 motifs was found to be elevated in AVITI reads.

This strand-specific drop in base quality in AVITI data also coincided with a rise in sequencing error rates at these loci (Fig. 4d). This supports the idea that G4 structures pose a challenge for avidity sequencing. Inspection of the soft-clipped portions suggested a non-random incorporation of bases into G4 motifs (Fig. 4a). Examination of mismatched bases in reads extending into G4 motifs revealed an excess of C-bases in the Element reads, a pattern not observed in Illumina data (Fig. 4e). This suggests that G4 motifs may hinder accurate cycle progression in AVITI sequencing, potentially leading to erroneous variant calls (Supplementary Fig. S8).

## Discussion

Comparing WGS results between PCR-free genome libraries sequenced on the AVITI and NovaSeq X Plus platforms, we found several platform-specific differences. The AVITI system provided lower duplication rates and also higher reported base qualities, which translated into higher mapping confidence and less spurious variant candidates. However, based on benchmarking against PacBio DeepVariant calls, overall variant-calling performance was highly comparable between the AVITI and NovaSeq X Plus platforms, except for INDELs at low coverages, where the AVITI performed better. Stratifying comparisons by genomic context revealed additional differences in the platforms. Looking at both genome coverage and variant-calling performance, AVITI demonstrated higher coverage and F1-scores than NovaSeq X Plus in GC-rich regions. The practical impact of this is likely to vary by application. While the effect might be marginal in human genomes with sufficient baseline coverage, it could prove advantageous in sequencing high-GC bacterial genomes or complex metagenomes. On the other end, AVITI showed reduced coverage, lower F1-score, and high error rate around GC homopolymeric regions. This may be related to difficulties in resolving G-quaduplex structures by the AVITI.

Optical duplicates consumed at best 12%–13% of reads with NovaSeq X Plus while being practically non-existent in AVITI. While optical duplicates increase sequencing costs, the current cost per base (based on list prices) for NovaSeq X/X Plus ($2.1/Gb) is about half that of AVITI ($5.6/Gb) and AVITI24 ($3.7/Gb). Pricing for high-volume purchases of AVITI reagents (requiring multiple instruments) rivals those of NovaSeq X/X Plus (∼$2/Gb), but in standard use it remains more expensive unless NovaSeq X/X Plus optical duplicates were to approach 50%. Still, the lack of optical duplicates could simplify AVITI data processing, likely removing the need for duplicate marking for PCR-free libraries.

Besides optical duplicates, another issue related to the amplification strategy employed in the NovaSeq X Plus is index hopping when multiplexing runs [50, 51]. Inspection of the AVITI CB FS found no evidence of index hopping, and to our knowledge, there have been no reports of such issues with AVITI, nor with other sequencing technologies that employ RCA-based amplification [52]. This absence of index hopping would make the AVITI platform particularly attractive for applications where even low-level multiplexing artifacts could compromise data integrity.

For small-variant benchmarking, we relied on orthogonal data in the form of PacBio DeepVariant calls, since no suitable small-variant truth set exists for the cell lines employed in this study. While this approach is less rigorous compared to other efforts [24, 27, 53], it should reliably detect major differences in variant-calling performance between the AVITI and NovaSeq X Plus. One limitation with using PacBio is the low performance around long homopolymers, which were not included in the analyzed high-confidence regions. Variant-calling performance around long homopolymers is of particular interest given the performance of AVITI sequencing in decoding these regions, as shown here and in other studies [5, 9, 43]. Furthermore, reliance on high-confidence regions risks overestimating performance by excluding difficult genome regions. For future studies, relying on resources such as the HG002 Q100 genome [54, 55] and the platinum pedigree [53] should help to resolve differences between the AVITI and NovaSeq X Plus in an even more comprehensive manner.

Investigations of sequencing error rates showed that NovaSeq X Plus displayed increasing error rates toward read ends, as expected for Illumina reads [40], while AVITI generally displayed more stable error rates. However, while AVITI error rates overall were lower in read 1, read 2 showed elevated error rates in the AVITI CB datasets. The latter was seemingly associated with shorter inserts in both our experiments and in some publicly available AVITI CB datasets. The major substitutions involved in this increased read 2 error rate, C>T/G>A and C>A/G>T, could be symptomatic of DNA damage through cytosine deamination and guanine oxidation. Altogether, this points to potential issues with the read turnaround process on the AVITI instrument, although the cause is currently unclear.

While AVITI CB and CB FS data performed comparably for most metrics assessed here, some differences were noted. CB FS inserts were slightly shorter and had, likely due to the lower flow cell occupancy, a higher base quality and associated overall lower error rate. Stratifying coverage by genomic context, AVITI CB FS had higher coverage in long (≥21 bp) non-GC homopolymers (HPs) and high-GC regions. Variant F1-scores were also higher in GC-rich regions and for INDELs in general, which may relate to the higher run base quality. AVITI CB had a higher error rate in read 2, especially for short fragments, compared to CB FS, but this pattern was only observed in part of the public AVITI datasets. While these performance differences are noteworthy, the conclusions we can draw are limited by our use of a single CB FS flow cell. Further investigation with a larger, controlled experimental design is necessary to determine if these shifts are inherent to the chemistry or specific to run conditions.

AVITI sequencing was found to be particularly affected by G-quadruplex motifs, with both pronounced base quality drops and increased sequencing errors following motifs on the template strand. The sequencing errors were mainly attributed to increased cytosine incorporation. This could be caused by polymerase stalling in the G4 guanidine repeats. Interestingly, this bias also presents an opportunity to effectively detect G4 motifs, as seen with native Illumina data [56] despite lower G4 formation under normal sequencing conditions [46]. Furthermore, G4 motifs in the template also increased quality in the bases preceding the motif (Fig. 4c), which we hypothesize reflects increased polony compaction. If true, this could prove a new avenue for future quality improvements for avidity sequencing.

Overall, we conclude that AVITI and NovaSeq X Plus produced highly comparable data for human WGS. However, each platform exhibits distinct strengths and limitations, in particular relating to genomic contexts and read error modes. When selecting a sequencing platform, these differences should be carefully considered in relation to the analyzed genome or application in question. While the lower cost per Gb might make the NovaSeq X more attractive for large-scale operations, the lower instrument cost of the AVITI allow individual labs to run WGS, along with an array of other omics assays.

## Supplementary Material

lqag053_Supplemental_File

## Data Availability

All analysis-related code including Snakemake workflows, configs, analysis scripts, and notebooks are available on GitHub (NationalGenomicsInfrastructure/NGI_Element_benchmark) and as a persistent copy on Zenodo (https://doi.org/10.5281/zenodo.17302039). Raw Element AVITI and Illumina NovaSeq X Plus sequencing data for all cell lines were submitted to European Nucleotide Archive (ENA), under the study accession PRJEB90663. PacBio Revio data for the multiple myeloma cell lines were uploaded to ENA under the study accession PRJEB95775.
