# Supplementary material for: Whole-genome sequencing with AVITI and NovaSeq X Plus reveals comparable performance with contextual biases
Source: NAR Genom Bioinform. 2026 May 26;8(2):lqag053. doi: 10.1093/nargab/lqag053 (PMC13202175; doi:10.1093/nargab/lqag053)
Supplement: lqag053_Supplemental_File [file lqag053_supplemental_file.pdf]

SUPPLEMENTARY TABLES AND FIGURES

Whole genome sequencing with AVITI and NovaSeq X Plus reveals comparable performance with contextual biases.

Pontus Höjer, Johannes Alneberg, Pär Lundin, Tom Martin, Julia Hauenstein, Helena Fällmar, Magnus Lindell, Christian Natanaelsson, Susana Häggqvist, Adam Ameur, Jessica Nordlund, Robert Månsson Welinder

Supplementary Table 1: Publicly available Element datasets used. CB = Cloudbreak, FS = Freestyle.

Dataset	Freestyle	File format	Link
Arslan et al. 2024 - HG002 (CB)	No	BAM	s3://avidity-manuscript-data/data/sentieon-bwa/20220601_PLT-03_BBS-0174-OBPA__FQD-2x150-35x/deduped.bam
GIAB - HG002 Long Insert (CB)	No	BAM	s3://giab/data/AshkenazimTrio/HG002_NA24385_son/Element_AVITI_20231018/HG002_GRCh38-GIABv3_Element-LngInsert_2X150_55x_20231018.bam
GIAB - HG002 Std Insert (CB)	No	BAM	s3://giab/data/AshkenazimTrio/HG002_NA24385_son/Element_AVITI_20231018/HG002_GRCh38-GIABv3_Element-StdInsert_2X150_81x_20231018.bam
Platinum Pedigree - NA12878 (CB)	No	BAM	s3://platinum-pedigree-data/data/element/mapped/GRCh38/2188-E.GRCh38.merged.sort.bam
GIAB - HG008-N-D Std Insert (CB)	No	BAM	s3://giab/data_somatic/HG008/Liss_lab/Element_AVITI_20240118/HG008-N-D_Element-StdInsert_61x_GRCh38-GIABv3.bam
Element Bio - HG002 (CB FS)	Yes	FASTQ	https://go.elementbiosciences.com/human-whole-genome-sequencing-third-party (JM-L825-HG002)

Supplementary Table 2: Duplication (optical and nonoptical) rate per lane for Illumina NovaSeq X Plus libraries.

Cell line	Lane 1 (140 pM)	Lane 2 (160 pM)
KMS12BM	27.7%	12.1%
MM1S	29.1%	12.8%
OPM2	28.9%	12.9%
REH	30.9%	13.5%

Supplementary Table 3: MM cell line PacBio Revio sequencing statistics.

Cell line	Base >= Q20 (%)	N50 fragment length (bp)	Mean coverage (X)
KMS12BM	97.2%	13,554	27.2
MM1S	97.3%	13,724	24.1
OPM2	97.3%	14,569	27.9

Supplementary Table 4: Extent of GIAB v3.5 homopolymer stratifications in GRCh38. GC-only refers to homopolymers with repeated G- or C-bases. Bases include 5 bp on each side of each repeat (e.g. slop).

Stratification	All bases (% of GRCh38)	GC-only bases (% of GRCh38)	% stratification GC-only
Homopolymers 7-11bp	49,788,855 (1.6%)	1,796,827 (0.1%)	3.61%
Homopolymers ≥12 bp	18,344,341 (0.6%)	80,541 (0.003%)	0.44%
Imperfect homopolymers ≥11 bp	44,462,862 (1.4%)	2,549,394 (0.1%)	5.73%

Supplementary Table 5: GIAB stratifications with significant overlap with pqsfinder predicted G4 motifs as computed with “bedtools fisher”. Only overlaps where at least 50% of bases were shared reciprocally were considered. P-values adjusted using Bonferroni method and only stratifications values below 0.01 kept. HP = homopolymer, Imp. HP = imperfect HP.

Stratification	Regions	Overlapping G4	% overlapping	Odds ratio
HP ≥21bp (GC)	66	64	97.0%	1,009.8
Imp. HP ≥21bp (GC)	3,439	2,993	87.0%	213.4
HP ≥12bp (GC)	3,398	2,673	78.7%	145.0
Imp. HP ≥11bp (GC)	107,713	77,378	71.8%	106.2
HP 7-11bp (GC)	101,792	30,204	29.7%	19.0
HP 4-6bp (GC)	12,048,931	613,434	5.1%	3.7
Imp. HP ≥11bp	1,678,630	76,546	4.6%	1.8
HP ≥7bp + Imp. HP ≥11bp	3,819,656	95,925	2.5%	1.0

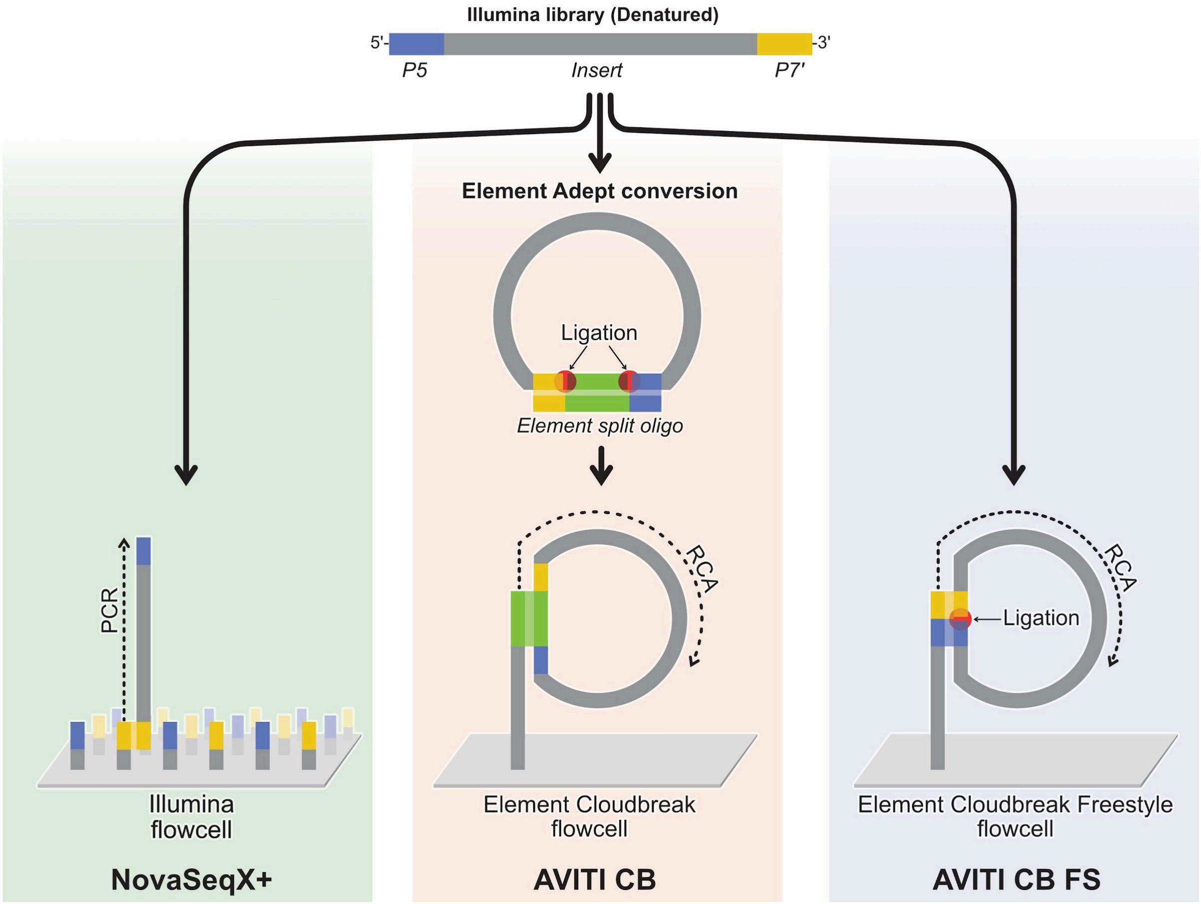

Supplementary Figure 1: Schematic illustration of the different Illumina NovaSeq X Plus and Element AVITI sequencing workflows utilized in the study. CB = Cloudbreak, FS = Freestyle.

a

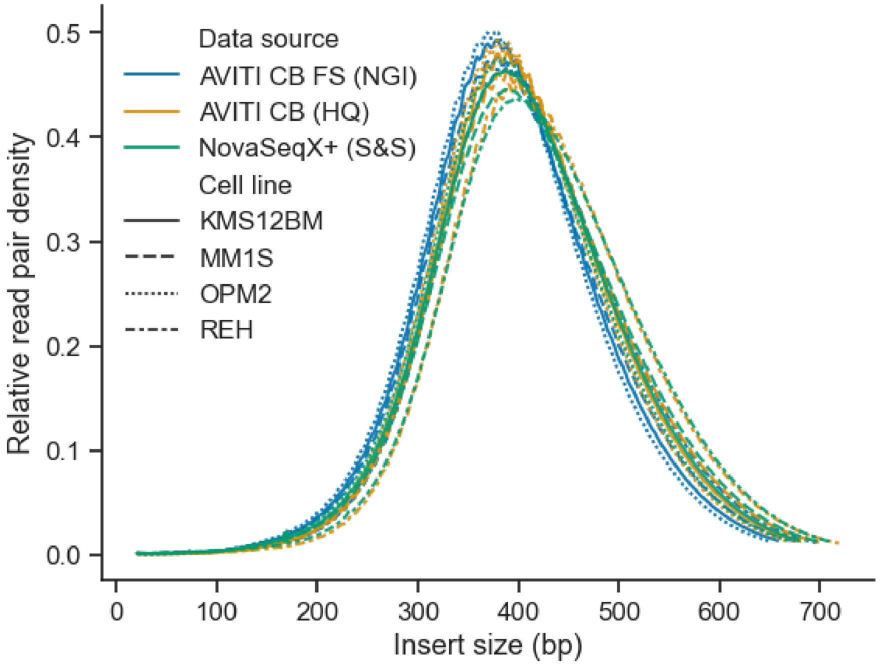

b

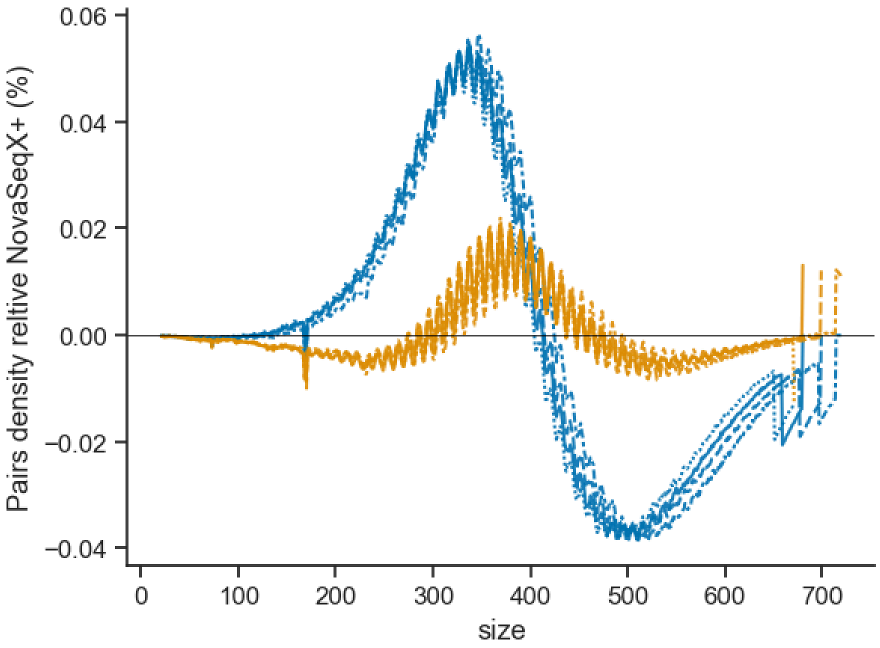

Supplementary Figure 2: Insert size distribution for the mapped libraries. (**a)** Read pair density by insert size. **(b)** Relative read pair density normalized for each cell line to NovaSeqX+.

a

b

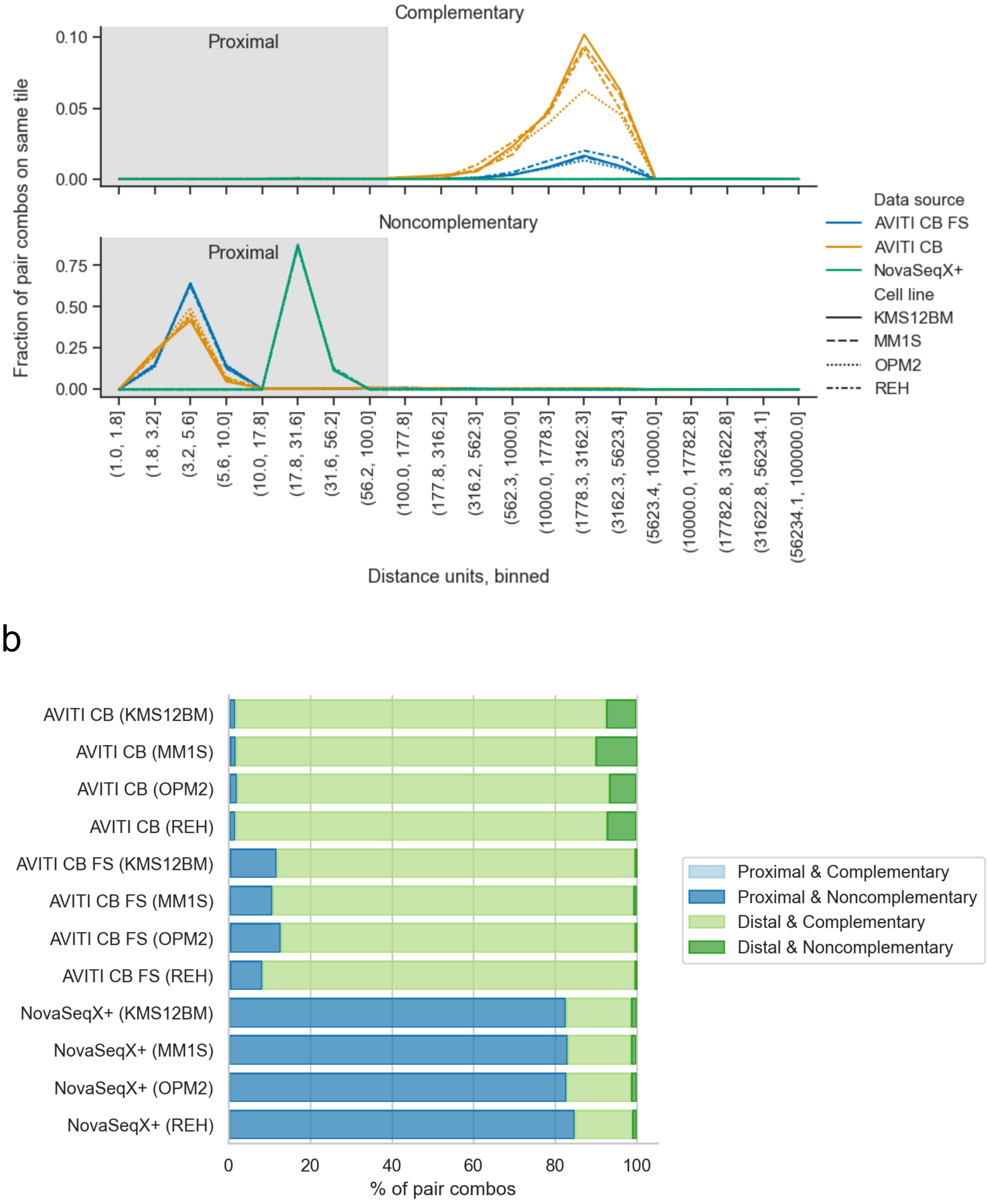

Supplementary Figure 3: Duplicate read pair classifications based on physical proximity on flow cell and complementarity. Pair combinations were classified as "Proximal" if their physical spacing was less than 100 distance units. Pair combinations were further classified as "Complementary" if the pairs were in opposite directions with regard to read1 and 2. (**a**) Pair density by spacing distance for pair combos on the same tile. (**b**) Pair classifications breakdown.

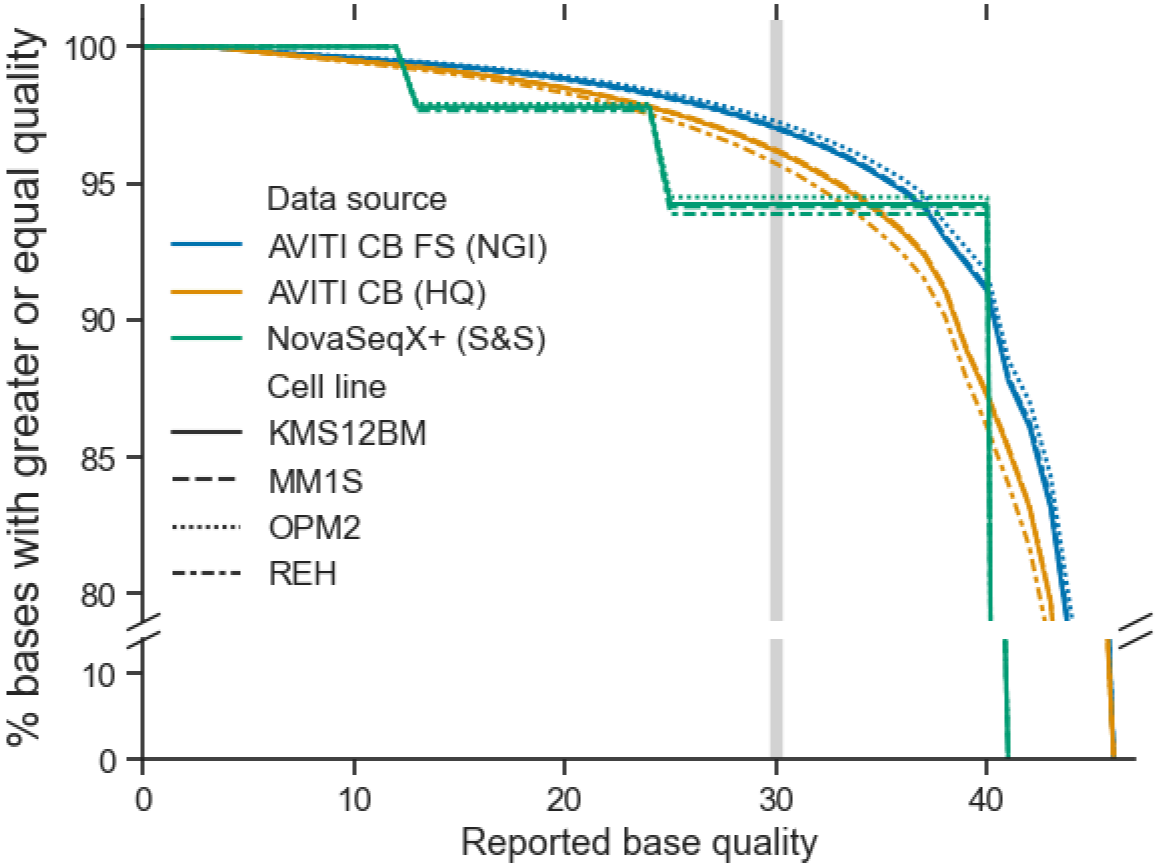

Supplementary Figure 4: Reported base quality distribution shown as the percentage of bases with a certain Q-score or greater. Illumina NovaSeq X Plus outputs Q-scores in four bins producing the stepped graph shown here. The highlighted area in grey indicates Q30.

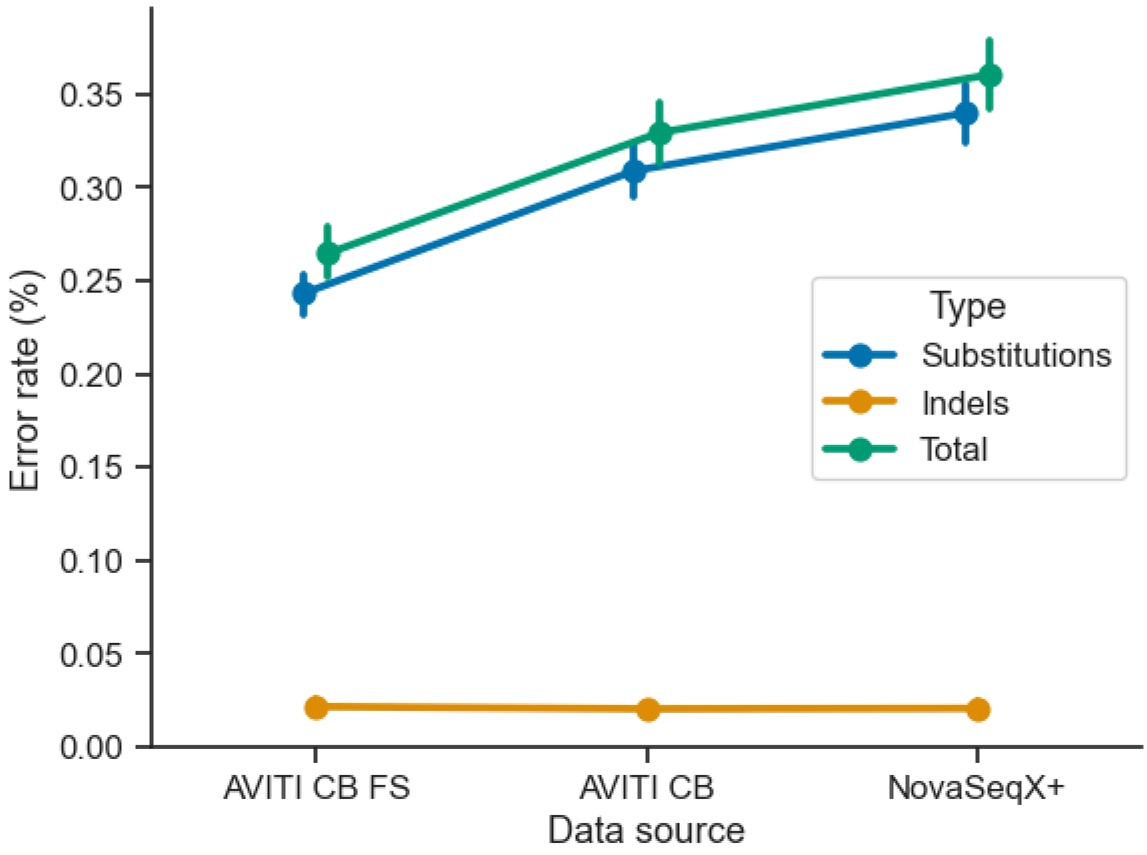

Supplementary Figure 5: Error rate as the percentage of bases mismatched to the reference is primarily driven by substitutions. Note that the mismatches include true variants.

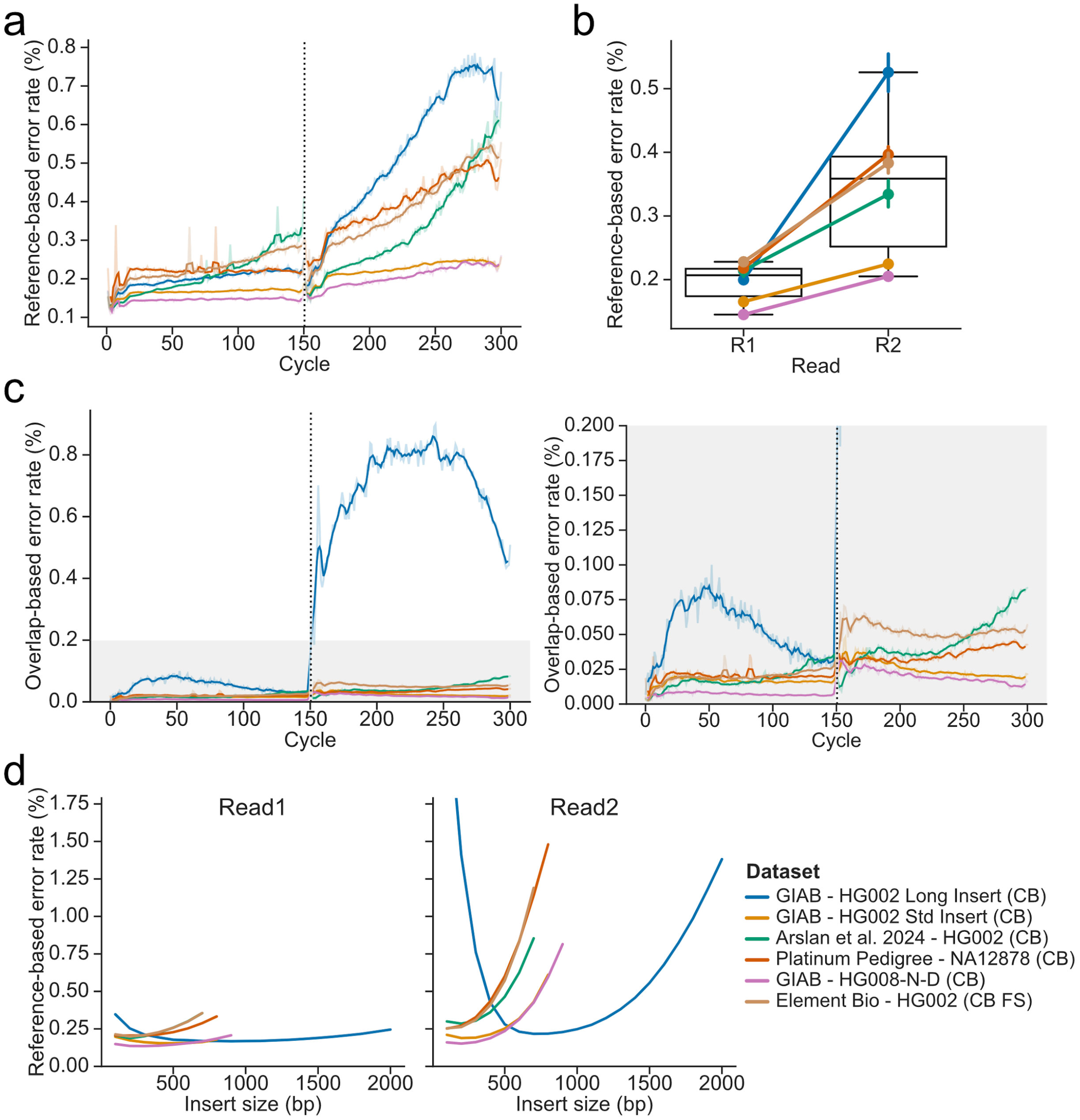

Supplementary Figure 6: Error rates in public Element AVITI datasets. (**a**) Error rate across cycles based on mismatches to the reference. The dotted line shows the boundary between reads 1 and 2. (**b**) Change in average error rate between read 1 and 2. (**c**) Error rate across cycles based read pair overlap mismatches. The right plot is a zoomed in version of the left plot within the area highlighted in grey. (**d**) Error rate across reads and insert sizes. Insert sizes calculated in 50 bp bins.

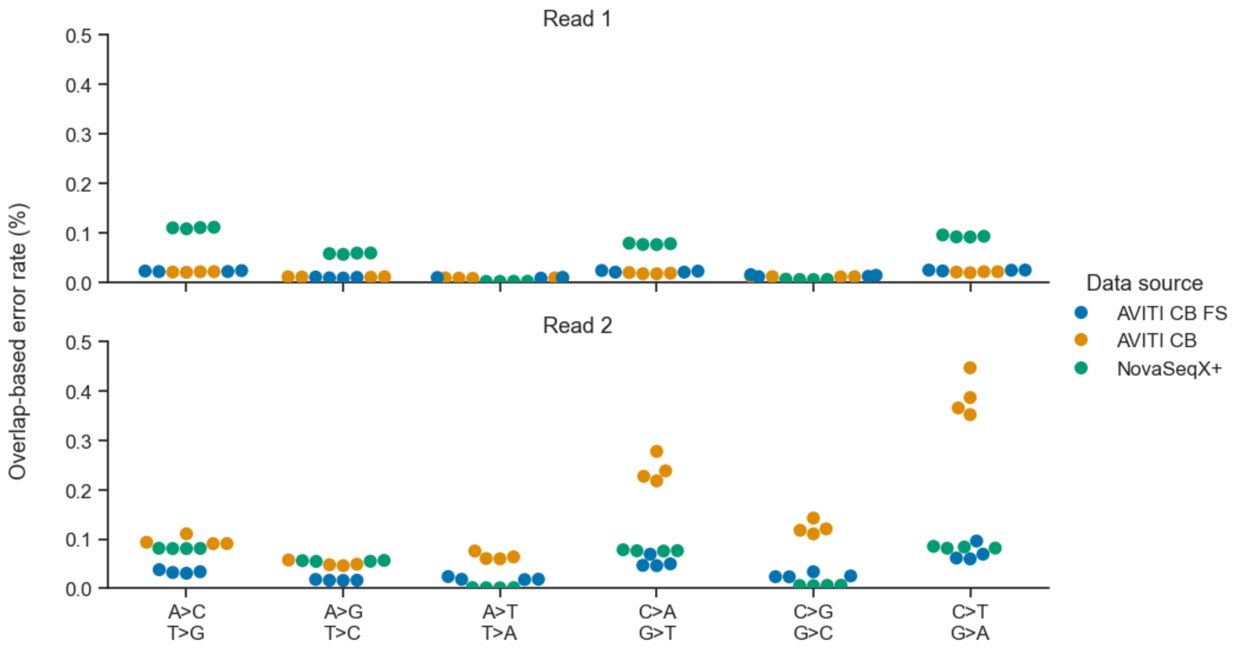

Supplementary Figure 7: Error rate by sequence context and read. Error rate calculated using fraguracy based on overlapping bases in read pairs. Note that only short fragments with an insert size <300 are included here.

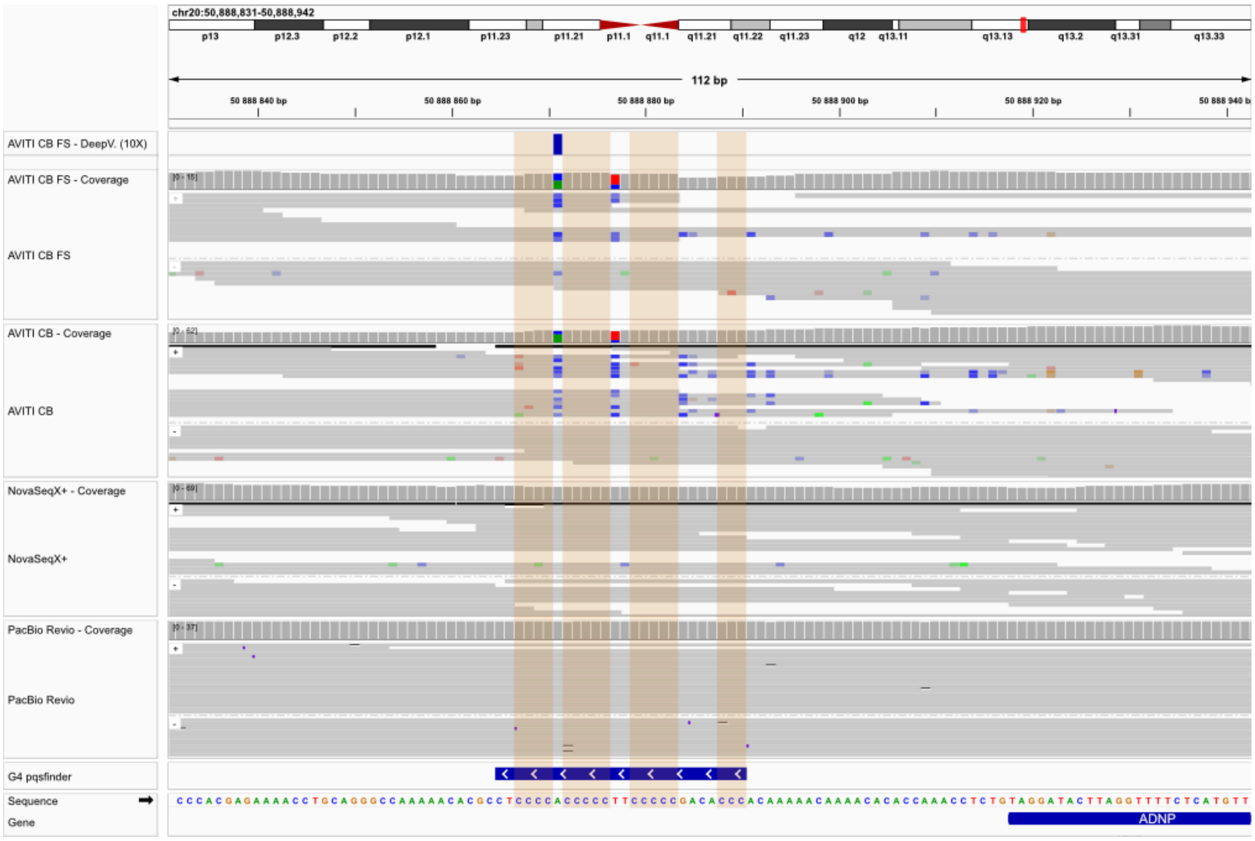

Supplementary Figure 8: Example of cysteine base-call errors in AVITI sequencing data related to G4 motif on opposing strand. Highlighted sections in orange show polyG stretches of G4 motif. Top row shows DeepVariant calls with AVITI CB FS at 10X coverage, generating a false positive substitution A>C in the G4-motif loop. Image shows data from MM1S cell line and was generated using IGV (v2.16.2) with soft-clipped bases hidden.
